# Neurokinin receptor mechanisms in forebrain medial septum modulate nociception in the formalin model of inflammatory pain

**DOI:** 10.1038/s41598-021-03661-6

**Published:** 2021-12-21

**Authors:** Si Yun Ng, Mohammed Zacky Ariffin, Sanjay Khanna

**Affiliations:** 1 Department of Physiology, Yong Loo Lin School of Medicine, National University of Singapore, MD9, 2 Medical Drive, Singapore, 117593 Singapore; 2 Neurobiology Programme, Life Sciences Institute, National University of Singapore, Singapore, Singapore; 3 Healthy Longevity Translational Research Programme, Yong Loo Lin School of Medicine, National University of Singapore, Singapore, Singapore

**Keywords:** Neural circuits, Sensorimotor processing, Sensory processing

## Abstract

The present study has explored the hypothesis that neurokinin1 receptors (NK1Rs) in medial septum (MS) modulate nociception evoked on hind paw injection of formalin. Indeed, the NK1Rs in MS are localized on cholinergic neurons which have been implicated in nociception. In anaesthetized rat, microinjection of L-733,060, an antagonist at NK1Rs, into MS antagonized the suppression of CA1 population spike (PS) evoked on peripheral injection of formalin or on intraseptal microinjection of substance P (SP), an agonist at NK1Rs. The CA1 PS reflects the synaptic excitability of pyramidal cells in the region. Furthermore, microinjection of L-733,060 into MS, but not LS, attenuated formalin-induced theta activation in both anaesthetized and awake rat, where theta reflects an oscillatory information processing by hippocampal neurons. The effects of L-733,060 on microinjection into MS were nociceptive selective as the antagonist did not block septo-hippocampal response to direct MS stimulation by the cholinergic receptor agonist, carbachol, in anaesthetized animal or on exploration in awake animal. Interestingly, microinjection of L-733,060 into both MS and LS attenuated formalin-induced nociceptive flinches. Collectively, the foregoing novel findings highlight that transmission at NK1R provide an affective valence to septo-hippocampal information processing and that peptidergic transmission in the septum modulates nociceptive behaviours.

## Introduction

The medial septum (MS), including septal cholinergic neurons, are implicated in nociception^1–7^. Indeed, septal cholinergic neurons project to the medial prefrontal cortex and the hippocampus, both of which are implicated in pain^1,8–13^. Further, hind paw injection of formalin, a model of inflammatory pain^14–18^, evokes an increase in extracellular level of acetylcholine in the hippocampus^19^. Conversely, selective destruction of septal cholinergic neurons attenuates formalin-induced (a) hippocampal theta, especially the power of theta, (b) suppression of amplitude of CA1 population spike (PS) and (c) the decrease in extracellular action potential discharge of pyramidal cells^3,5,14,15^. Furthermore, intra-hippocampal administration of atropine, a cholinergic muscarinic receptor antagonist, attenuated the noxious stimulus-induced suppression of CA1 PS^20^. Collectively, the preceding suggests that the hind paw injection of formalin excites septal cholinergic neurons to modulate CA1 pyramidal cell nociceptive responses by intrahippocampal release of acetylcholine. Here, it is notable that the amplitude of PS reflects the synaptic excitability of population of CA1 pyramidal cells, while the power of theta reflects the population size of neurons synchronized into 3–12 Hz theta rhythmic activity during information encoding. The theta provides a basis for temporal organization of information in the hippocampus^21–23^.

Pharmacological investigations show that septal glutamate transmission at AMPA and NMDA receptors mediate nociception, including in the formalin model^2,24^. However, little else is known of septal pharmacology in relation to nociception. Interestingly, within the MS, the neurokinin receptors (NKRs) are expressed almost exclusively in the septal cholinergic neurons^25–29^. Indeed, agonists at NKRs, including substance P (SP), excite cholinergic neurons. While the septal cholinergic neurons modulate hippocampal nociceptive processing (see above), the physiological role of septal NKRs in nociception is not known at present. Interestingly, NKRs elsewhere in CNS are implicated in noxious information processing. For instance, hind paw injection of formalin elevates both spinal immunoreactivity for SP and the level of SP in spinal dialysates^30–33^. Conversely, formalin-induced nociceptive responses are attenuated on administration of NKR antagonists or on disruption of SP neurotransmission with a variety of molecular and cellular methodologies, especially in the spinal cord and the rostral ventrolateral medulla^34–36^. Based on the preceding, we hypothesized that NK1Rs in medial septum (MS) mediates septo-hippocampal nociceptive processing and nociceptive behaviours in the formalin model. Here it is notable that hind paw injection of formalin evokes strong and persistent overt nociceptive behaviours and robust electrophysiological changes leading to an extensive use of the model for exploring physiological/pharmacological basis of acute nociception.

## Results

### Pharmacological investigations in anaesthetized rat

**Figure 1:**
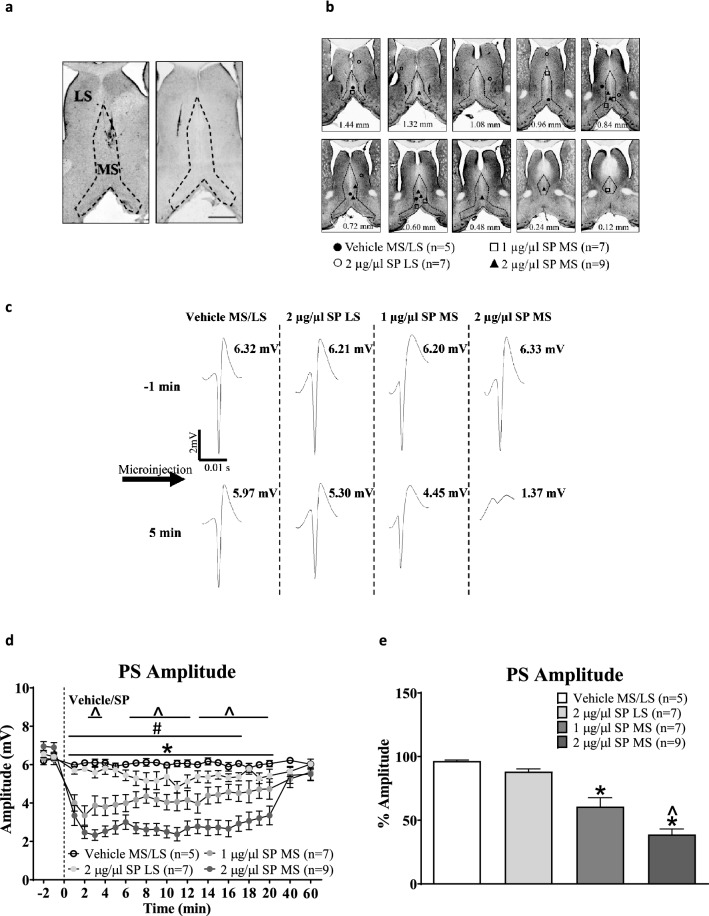
Microinjection of Substance P (SP) into medial septum (MS) evokes a dose-dependent suppression of the amplitude of CA1 population spike (PS) in anaesthetised animals. (**a**) Representative microinjection site in the MS (left) or the lateral septum (LS, right). The microinjection site was identified by the needle track and/or observation of the dye spot. Demarcation between MS and LS was based on the atlas of rat brain by Paxinos and Watson (2007). Scale bar represents 1 mm. (**b**) Composite of microinjection sites in the septal region. SP (1 or 2 µg/µl, 0.5 µl) or the corresponding vehicle was microinjected into either MS or LS via a single 33G microinjection stainless steel needle coupled to a microsyringe. Number at bottom of each panel represents distance from Bregma. Note the distribution of microinjection sites along the anterior–posterior and medio-lateral extent of the septal region. (**c**) The PS traces depicting the representative PS before (-1 min) and after (5 min) SP microinjection. Numbers next to each trace reflect the PS amplitude. Arrow indicates microinjection of SP. (**d**) Time course of the effect of intraseptal SP on the amplitude of CA1 PS. A given concentration of SP (or vehicle) was microinjected thrice with at least 1 h between microinjections. Since the effect of repeated injections at a given dose (1 or 2 µg/µl) and at the selected site (MS or LS) were comparable, an average response for that dose and site was built by averaging the time course for the three microinjections in a given experiment and then for the entire group. Time of microinjection is given by the dashed vertical line at 0 min. (**e**) Histogram illustrating the average amplitude of PS in first 5 min after microinjection expressed as a percentage of the control PS amplitude (average of PS amplitude in − 2 and − 1 min). Data are mean ± S.E.M. Significant difference (p < 0.05): (**d**) *‘2 µg/µl SP MS’ group vs. ‘Vehicle MS/LS’ group; ^#^‘1 µg/µl SP MS’ group vs. ‘Vehicle MS/LS’ group; ^‘2 µg/µl SP MS’ group vs. ‘2 µg/µl SP LS’ group; two-way RM ANOVA followed by Bonferroni post-hoc test. (**e**) *vs. Vehicle MS/LS; ^vs. 2 µg/µl SP LS; Kruskal–Wallis test followed by Dunn’s post-hoc test.

**Figure 2:**
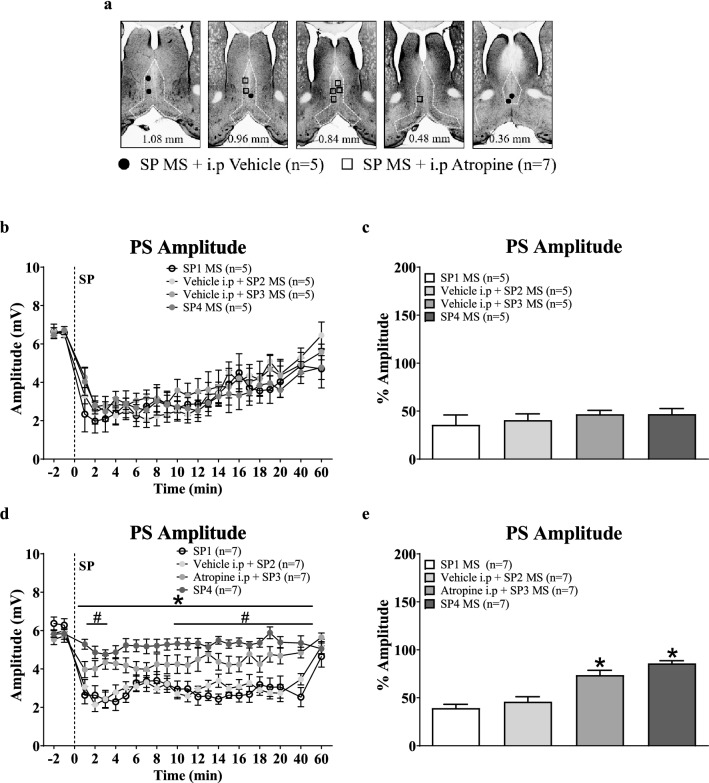
Intraperitoneal administration of atropine blocked Substance P (SP)-induced suppression of CA1 population spike (PS) in anaesthetised animals. SP (2 µg/µl, 0.5 µl) was microinjected into the MS 4 times (SP1, SP2, SP3, SP4) with at least 1 h between microinjections. The animals were injected intraperitoneally (i.p.) with vehicle or atropine (5 mg/kg) half an hour prior to SP2 and SP3 microinjections. Time course plots and histograms are built as in Fig. 1. (**a**) Composite of microinjection sites in the MS, built as in Fig. 1. (**b**, **d**) Time course graphs illustrating the effect of systemic administration of vehicle (**b**) or atropine (**d**) on the suppression of CA1 PS induced by intra-MS SP. (**c**, **e**) Histogram illustrating the average amplitude of PS in first 5 min after microinjection expressed as a percentage of the control PS amplitude (average of PS amplitude in − 2 and − 1 min). Note that administration of atropine (**d**, **e**), but not vehicle (**b**, **c**), blocked the suppression induced by microinjection of SP into the MS. Data are mean ± S.E.M. Significant difference (p < 0.05): (**d**) ^#^Atropine i.p + SP2 MS vs. SP1, *SP4 MS vs. SP1 MS; two-way RM ANOVA followed by Bonferroni post-hoc test. (**e**) *vs.SP1 MS; one-way ANOVA followed by Newman–Keuls post-hoc test.

**Figure 3:**
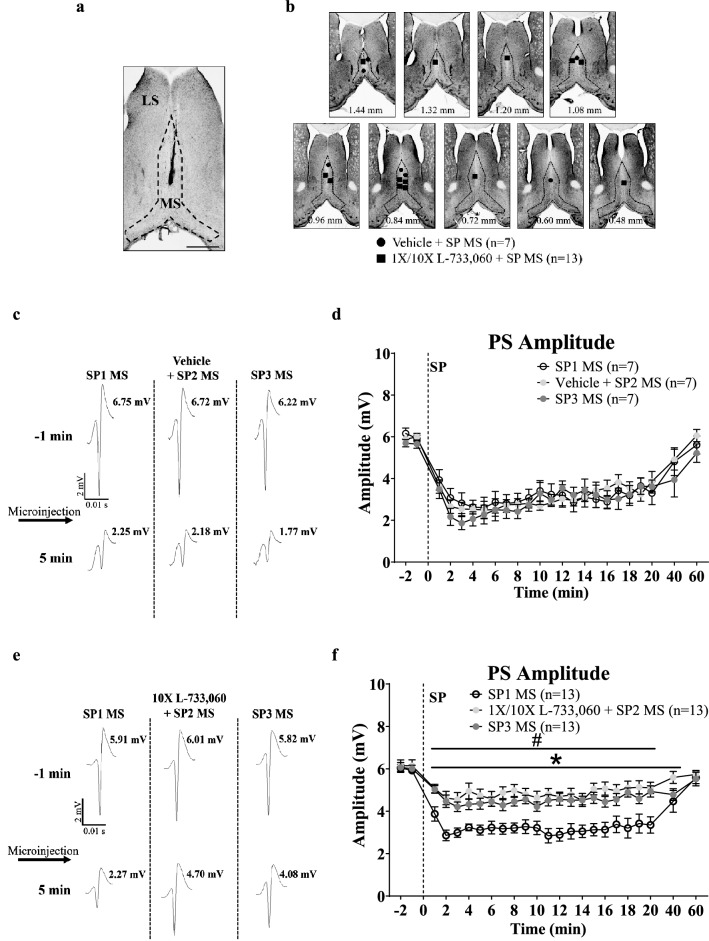
Microinjection of neurokinin 1 receptor (NK1R) antagonist, L-733,060, into the medial septum (MS) blocked Substance P (SP)-induced suppression of CA1 population spike (PS) in anaesthetised animals. A double-barrelled cannula was used for microinjection of the drugs. SP (2 µg/µl, 0.5 µl; posterior cannula) was microinjected three times (SP1, SP2, SP3), at least 1 h apart, with vehicle or L-733,060 (1 ×, 0.0176 µg/µl; 10 ×, 0.176 µg/µl, 0.5 µl; anterior cannula) microinjected 15 min before SP2. (**a**) Representative microinjection site in the MS. Scale bar represents 1 mm. (**b**) Composite of microinjection sites in the MS built as in Fig. 1. (**c**, **e**) The PS traces representing the average PS before (− 1 min) and after (5 min) SP microinjection. Numbers next to each trace reflects the computed PS amplitude. Arrow indicates microinjection of SP. (**d**, **f**) Time course of effect of pre-treatment with vehicle (**d**) or L-733,060 (**f**) on SP-induced suppression of PS. Notice the robust decrease in amplitude of PS amplitude with repeat microinjection of SP in vehicle pre-treated animal (**c**, **d**) which is attenuated on pre-treatment with the antagonist (**e**, **f**). Data are mean ± S.E.M. Significant difference (p < 0.05): (**f**) *1 ×/10 × L-733,060 + SP2 MS vs. SP1, ^#^SP3 vs. SP1 MS; two-way RM ANOVA followed by Bonferroni post-hoc test.

A series of experiment were performed in anaesthetized animal to explore the role of septal NK1Rs in modulating hippocampal neural activity. These experiments included:

- Identifying an effect of intraseptal SP on hippocampal neural activity and investigating whether the SP-induced change is antagonized by systemic atropine, a cholinergic muscarinic-receptor antagonist (Figs. 1 and 2). Since substance P (SP) excites septal cholinergic neurons, we anticipated that intraseptal SP-induced neural response in hippocampus is sensitive to antagonism by the cholinergic receptor antagonist,
- Testing whether SP-induced change is sensitive to antagonism by the NK1R antagonist, L-733,060 (Fig. 3),
- Exploring the pharmacological selectivity of L-733,060 in antagonizing the effect of intraseptal SP vs. intraseptal carbachol. Carbachol is a cholinergic receptor agonist and evokes robust septo-hippocampal activation, and
- Investigating the effect of intraseptal L-733,060 on formalin-induced hippocampal neural responses.

Two electrophysiological parameters were recorded during the experiments, namely CA1 PS and local theta wave activity. The findings in context of PS and theta wave activity are reported separately to facilitate presentation.

### Population spike (PS)

The PS was evoked every 10 s (i.e. at frequency of 0.1 Hz) on stimulation of field CA1. The amplitude of the PS correlates with number of CA1 pyramidal cells excited synaptically on stimulation of the Schaffer/commissural collaterals in field CA3.

#### Dose dependent effect of SP microinjection on CA1 PS (Fig. 1a–d)

In separate experiments, SP (1 µg/µl, n = 7; 2 µg/µl, n = 9) or vehicle (n = 5) was microinjected thrice, at least 1 h apart, into MS or LS (Fig. 1a,b). Since the effect of repeated injections at a given dose and at the selected site was comparable, an average response for that dose and site was built by averaging the time course for the three microinjections in each experiment. Statistical analysis of the time course revealed that microinjection of SP into MS, but not LS, evoked a robust suppression of PS for 20 min as compared to vehicle treatment (Fig. 1c; Treatment, F_3, 552_ = 17.76, p < 0.0001; two-way RM ANOVA followed by Bonferroni post-hoc test). SP at the higher dose of 2 µg/µl induced a more sustained and stronger effect when compared to the lower dose of 1 µg/µl (Fig. 1c). Indeed, comparison of the average PS amplitude in the first 5 mins after drug microinjection, expressed as percentage of the ‘control amplitude’, indicated that the suppression evoked on microinjection of the higher dose of SP into MS was significantly stronger as compared to the lower dose (Fig. 1d; Groups, p < 0.0001; Kruskal–Wallis test followed by Dunn’s post-hoc test). The ‘control amplitude’ was the average amplitude of the PS in the 2 min period (− 2 and − 1 min time points) before microinjection of SP.

#### Effect of atropine on SP induced response (Fig. 2a–e)

In the experiment, SP was microinjected 4 times (SP1-4), at least one hour apart. Vehicle or atropine were administered intraperitoneally 30 min prior to the microinjection of SP2 or SP3 (Fig. 2a).

Vehicle pre-treatment had no effect on intraseptal SP-induced suppression of CA1 PS (Fig. 2b; Treatment, F_3, 368_ = 0.26, p > 0.8; two-way RM ANOVA) which was, however, attenuated by pre-treatment with atropine (Fig. 2d; Treatment, F_3, 552_ = 16.31, p < 0.001; two-way RM ANOVA followed by Bonferroni post-hoc test). Indeed, the average percentage of PS amplitude was very similar across the repeated microinjections of SP in animals pre-treated with vehicle (Fig. 2c; Groups, F_3, 16_ = 0.58, p > 0.6; one-way ANOVA). In contrast, the SP-mediated decrease in average percentage of PS amplitude was attenuated with atropine pre-treatment (Fig. 2e; Groups, F_3, 24_ = 26.19, p < 0.0001; one-way ANOVA followed by Newman–Keuls post-hoc test).

#### Effect of intraseptal L-733,060 on SP-induced response (Fig. 3a–f).

This experiment examined whether the effect of intraseptal SP was sensitive to antagonism by the NK1R antagonist, L-733,060. The antagonist (0.0176 µg/µl, 1 × or 0.176 µg/µl, 10 ×) or the vehicle was microinjected into MS 15 min before SP2 (Fig. 3a,b).

SP evoked a robust and comparable suppression across repeat microinjections (SP1-SP3) into MS in control animals pre-treated with vehicle (Fig. 3c,d; Treatment, F_2, 414_ = 0.28, p > 0.7; two-way RM ANOVA). In contrast, two-way RM ANOVA followed by Bonferroni post-hoc test showed a significant effect of pre-treatment with 1 × (Treatment, F_2, 441_ = 5.38, p < 0.02, n = 8) and 10 × (Treatment, F_2, 252_ = 23.62, p < 0.0001, n = 5) dose of L-733,060 on SP-induced suppression. The time courses of SP-induced change in the amplitude of CA1 PS were very similar with two doses of the antagonist (1 × vs. 10 ×; SP1, Treatment, F_1, 253_ = 4.80, p > 0.05; SP2, Treatment, F_1, 253_ = 0.40, p > 0.5; SP3, Treatment, F_1, 253_ = 1.32, p > 0.2; two-way RM ANOVA; data not shown). Thus, the two groups were combined (1 ×/10 × L-733,060, n = 13). A comparison of the time courses of effect of repeat microinjections of SP in the combined group revealed an attenuation of suppression of PS amplitude at SP2 and SP3 compared to SP1 (Fig. 3e,f; Treatment, F_2, 828_ = 14.87, p < 0.0001; two-way RM ANOVA followed by Bonferroni post-hoc test).

#### Lack of effect of L-733,060 on basal amplitude

In this context, the basal amplitude refers to the average PS amplitude in the 2 min period (i.e., − 2 min and − 1 min time points) before microinjection of SP (SP1-3) in the experiments illustrated in Fig. 3. The basal amplitude before SP2 also corresponds to average amplitude of PS in the 14th and 15th minute after microinjection of either vehicle or L-733,060. In the Vehicle pre-treated groups, the basal amplitude of PS prior to SP2 was no different from the basal amplitudes prior to SP1 and SP3 microinjections in the group (Groups, F_2, 18_ = 2.29, p > 0.06; one-way ANOVA; data not shown). Similarly, L-733,060 pre-treatment did not affect the basal amplitude of the PS prior to SP2 as compared to basal amplitudes prior to SP1 and SP3 microinjections (Groups, F_2, 36_ = 0.16, p > 0.8; one-way ANOVA; data not shown). The basal amplitudes of the PS preceding each SP microinjection were also compared across groups. For example, the basal amplitude of the PS preceding SP1 microinjection in the vehicle pre-treated group was compared with the corresponding basal amplitude prior to SP1 microinjection in the L-733,060 pre-treated group. Such comparison indicated that basal amplitudes of the PS prior to each microinjection of SP in the vehicle pre-treated group were similar to the corresponding basal amplitudes of PS in the L-733,060 pre-treated group (SP1, p > 0.8; SP2, p > 0.6; SP3, p > 0.2; two-tailed unpaired t-test; data not shown).

#### Lack of effect of L-733,060 on carbachol-induced suppression of PS (Fig. 4a–e)

**Figure 4:**
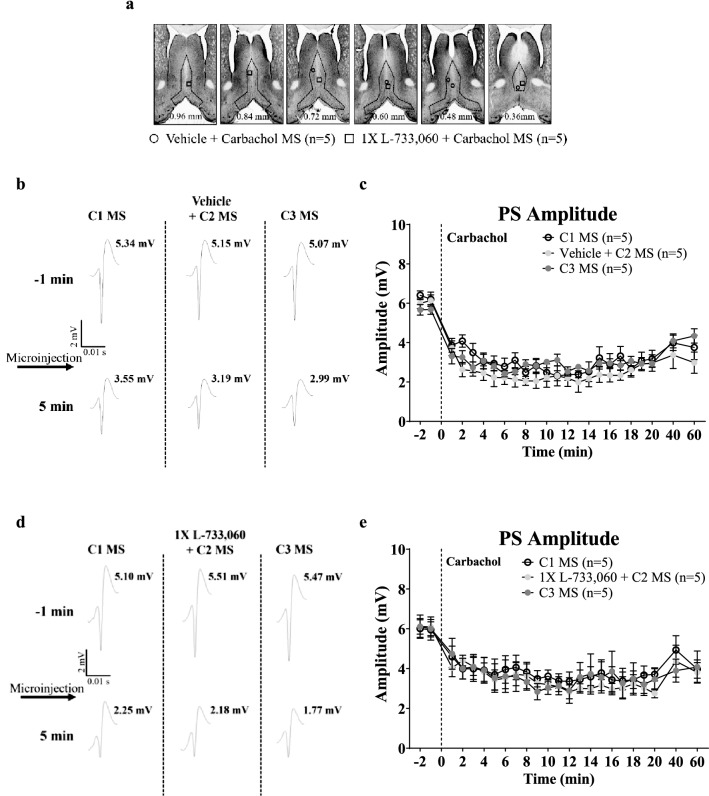
Microinjection of L-733,060 into the medial septum (MS) does not affect carbachol-induced suppression of CA1 PS in anaesthetized animals. (**a**) Composite of the microinjection sites in the MS was built as in Fig. 1. Carbachol (0.156 µg/µl, 0.5 µl) was microinjected three times (C1, C2 and C3), at least 1 h apart, with vehicle or L-733,060 (1 ×, 0.0176 µg/µl, 0.5 µl) microinjected 15 min before C2. (**b**, **d**) Representative PS traces following repeat carbachol microinjection into the MS. (**c**, **e**) Time course of change in amplitude of CA1 PS on repeat microinjection of carbachol. Note that carbachol elicited robust PS amplitude suppression which was unaffected by pre-treatment with vehicle or L-733,060. Data are presented are mean ± S.E.M.

The experiment was performed to evaluate the pharmacological selectivity of L-733,060 in antagonizing the responses to SP. To this end, we evaluated the effectiveness of L-733,060 in antagonizing the neural response to microinjection of carbachol, a cholinergic receptor agonist, into MS. Microinjection of carbachol into MS evoked a suppression of CA1 PS that was similar to that seen with intraseptal SP (see below). The protocol followed was the same as above. During the experiment, carbachol was microinjected thrice (C1–3), each microinjection being at least 1 h apart. The NK1R antagonist, L-733,060, or vehicle was microinjected 15 min prior to C2. Here it is notable that in the experiment described above the lower and the higher doses of the antagonist, L-733,060, were found to be equally effective in antagonizing the responses to SP. On this basis, we selected only one dose of the antagonist (i.e., 1 ×) as a representative dose to evaluate the selectivity of the antagonist.

In vehicle pre-treated animals, a robust suppression of CA1 PS was evoked with each microinjection of the agonist into MS (Fig. 4a–c; C1: Time, F_23, 92_ = 14.42, p < 0.0001; vehicle + C2: Time, F_23, 92_ = 14.75, p < 0.0001; C3: Time, F_23, 92_ = 11.69, p < 0.0001; n = 5, one-way RM ANOVA followed by Newman–Keuls post-hoc test). The time courses of change in PS amplitudes from 2 min before to 60 min after carbachol microinjections were comparable across the repeat microinjections suggesting a lack of effect of the vehicle pre-treatment on carbachol-induced suppression (Fig. 4b,c; Treatment, F _2, 276_ = 1.513, p > 0.2; two-way RM ANOVA). Likewise, carbachol induced a robust suppression on repeat microinjections into MS in L-733,060 (1 ×) pre-treated animals, the time courses of change in amplitudes being similar across the repeat microinjections of carbachol (Fig. 4d,e; Treatment, F_2, 276_ = 0.13, p > 0.8, two-way RM ANOVA).

#### SP and carbachol induced similar suppression of PS

This section compares the suppression evoked by the two agonists, i.e., SP and carbachol. A comparison was also made of the basal amplitudes of the PSs against which the suppression was induced on microinjection of the agonists. The comparisons were performed to discount the possibility that the differences in the effectiveness of L-733,060 in antagonizing the neural responses to SP vs. carbachol was due to differences in amplitudes of the basal PS preceding drug microinjection. To this end, the time courses of change with SP1 microinjections in the vehicle and the L-733,060 pre-treatment groups were combined (n = 20). Likewise, for C1 microinjections (n = 10). Two-way RM ANOVA of the time course of change evoked by SP1 vs. C1 revealed an insignificant effect of treatment (Treatment, F_1, 644_ = 0.03, p > 0.8; data not shown), suggesting that PS suppression evoked by the two agonists was similar. The control amplitudes of PS preceding microinjections were also similar (SP1 vs. C1: 6.05 ± 0.13 mV vs. 6.09 ± 0.24 mV, t = 0.1639, p > 0.8; two-tailed unpaired t-test).

#### Effect of microinjection of L-733,060 on the suppression of CA1 PS induced on hind paw injection of formalin (Fig. 5a–c)

**Figure 5:**
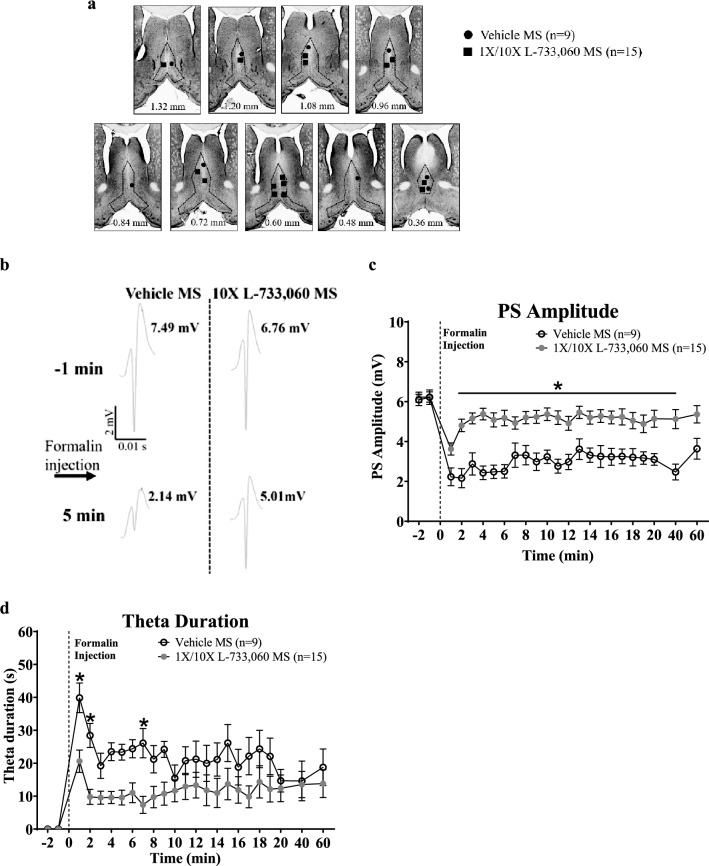
Microinjection of L-733,060 into the medial septum (MS) attenuated formalin-evoked suppression of CA1 PS and theta activation in anaesthetized animals. Formalin (5%, 0.05 ml) was injected subcutaneously into right hind paw 15 min after intraseptal microinjection of vehicle (n = 9) or the NK1R antagonist, L-733,060 (1 ×, 0.0176 µg/µl, n = 8; 10 ×, 0.176 µg/µl, n = 7) during period of large irregular hippocampal field activity (LIA). (**a**) Composite representation of microinjection sites in MS built as in Fig. 1. (**b**) Microinjection of L-733,060 into MS attenuated formalin-induced suppression of CA1 PS. The PS traces are built as in Fig. 3. (**c**, **d**) Time course of effect of pre-treatment with vehicle or L-733,060 on SP-induced suppression of PS (**c**) and duration of theta wave activity (**d**). The duration of theta reflects the cumulative duration of theta segments of at least 1 s length in blocks of 1 min. Data are mean ± S.E.M. Significant difference (p < 0.05): * vs. Vehicle MS; two-way RM ANOVA followed by Bonferroni post-hoc test.

Hind paw injection of formalin in the anaesthetised animals is known to induce a suppression of CA1 PS^3^. The experiment in this study was performed to investigate if the formalin-induced suppression is sensitive to antagonism by intraseptal L-733,060. During the experiment, formalin was injected 15 min after pre-treatment with L-733,060 (1 × or 10 ×) or vehicle on microinjection into the MS (Fig. 5a–c). Consistent with the previous observations^3^, injection of formalin evoked a robust and sustained suppression of the amplitude of CA1 PS in vehicle pre-treated control experiments (Fig. 5b; Time, F_23, 184_ = 6.40, p < 0.0001; one-way RM ANOVA followed by Newman Keuls post-hoc test, n = 9). A significant suppression was observed to 60th min after formalin injection.

However, intraseptal L-733,060 (1 × or 10 ×) attenuated the suppression of CA1 PS induced on formalin injection (Fig. 5b,c). Statistical analyses indicated an overlapping effects of 1 × L-733,060 (n = 8) and 10 × L-733,060 (n = 7) on the time course of formalin-induced suppression (Treatment, F_1, 299_ = 0.29, p > 0.5; two-way RM ANOVA). Thus, the two groups were combined (‘1 ×/10 × L-733,060 MS group’, n = 15) and compared with the vehicle treated group. In this context, two-way RM ANOVA showed a significant effect of L-733,060 pre-treatment (Fig. 5b,c; Treatment, F_1, 506_ = 25.10, p < 0.0001), while Bonferroni post-hoc test showed that suppression of PS amplitude was attenuated with L-733,060 pre-treatment along the time course monitored in the experiment.

### Theta wave activity

#### Effect of L-733,060 microinjection on theta activity in anaesthetised animal (Fig. 5d)

Hippocampal theta field activity was recorded concurrently with PS in the preceding experiments. Interstingly, while the SP-induced suppression of CA1 PS was immediate and robust, the theta activation was weak. In this context, the average theta duration/min in the first 5 min after microinjection of SP into MS was not significantly different from the control experimental groups (Vehicle vs. SP (2 µg/µl) in LS vs. SP (1 µg/µl) in MS vs. SP (2 µg/µl) in MS, 1.49 ± 0.76 s (n = 5) vs. 2.91 ± 0.85 s (n = 7) vs. 4.64 ± 1.47 s (n = 7) vs. 8.26 ± 2.16 s (n = 9); Groups, p > 0.1; Kruskal–Wallis test). In the combined group that included responses to all SP1 microinjections, the average duration of theta per min within the first 5 min was also low (7.44 ± 2.16 s/min, n = 20). On the other hand, carbachol microinjection into the MS elicited a relatively robust increase in the average duration of theta in the 5 min after C1 (26.74 ± 2.95 s/min, n = 10; time course data not shown). Thus, the effect of L-733,060 on theta frequency and theta power was analyzed only on carbachol-induced theta.

Statistical analysis revealed a lack of significant effect of L-733,060 pre-treatment on carbachol-induced theta frequency (Groups, F_2, 12_ = 1.12, p > 0.3, n = 5; one-way ANOVA), normalised theta power (Groups, F_2, 12_ = 0.92, p > 0.4, n = 5; one-way ANOVA) and the average duration of theta per min (Groups, F_2, 12_ = 1.33, p > 0.3, n = 5; one-way ANOVA). In this context, the FFT theta peak frequencies with C1, L-733,060 + C2 and C3 were 4.94 ± 0.16 Hz, 4.50 ± 0.26 Hz and 4.32 ± 0.30 Hz, respectively. The corresponding values of normalized FFT theta power were 0.42 ± 0.06, 0.53 ± 0.07, and 0.51 ± 0.09. While the values of theta duration corresponding to the three repeat microinjections were 21.73 ± 3.93 s vs. 21.42 ± 7.20 s and 18.87 ± 6.94 s.

With regards to the formalin test, a relatively robust theta activation was observed after hind paw injection of formalin (Fig. 5d). Statistical analysis revealed that L-733,060 pre-treatment significantly attenuated the duration of theta induced on hind paw injection of formalin (Fig. 5d; Treatment, F_1, 462_ = 6.36, p < 0.02; two-way RM ANOVA followed by Bonferroni post-hoc test). Consistently, the average duration of theta per min in the first 5 min following injection of formalin was also attenuated (‘Vehicle MS’, n = 9 vs. ‘L-733,060 MS’, n = 15, 26.88 ± 2.29 s vs. 11.82 ± 1.91 s; p < 0.0001; two-tailed unpaired t-test). However, normalised theta power (‘Vehicle MS’, n = 9 vs. ‘L-733,060 MS’, n = 15, 0.66 ± 0.10 vs. 0.55 ± 0.07; p > 0.3; two-tailed unpaired t-test) and theta frequency (‘Vehicle MS’, n = 9 vs. ‘L-733,060 MS’, n = 15, 3.86 ± 0.16 Hz vs. 3.76 ± 0.10 Hz; p > 0.6; two-tailed unpaired t-test) were unaffected.

Since theta power was normalized to spontaneous theta, we compared the parameters of spontaneous theta recorded at the beginning of each experiment. In the study involving carbachol microinjections, the power of spontaneous theta was not different between the experimental groups (vehicle, 0.05 ± 0.01 mV^2^ (n = 5) vs. L-733,060, 0.04 ± 0.01 mV^2^ (n = 5), p > 0.5; two-tailed unpaired t-test). The FFT theta peak frequency of spontaneous theta was also not different between the two groups (vehicle, 4.20 ± 0.20 Hz (n = 5) vs. L-733,060, 3.80 ± 0.12 Hz (n = 5), p > 0.1; two-tailed unpaired t-test).

In the formalin study, the FFT theta peak power of spontaneous theta in the groups pre-treated with vehicle (n = 9) or L-733,060 (n = 15) was not different from each other (0.07 ± 0.01 mV^2^ vs. 0.05 ± 0.006 mV^2^, t = 2.038, p > 0.05; two-tailed unpaired t-test). Likewise, the frequencies were also not different (vehicle, n = 9 vs. L-733,060, n = 15, 4.18 ± 0.20 Hz vs. 4.00 ± 0.010 Hz, p > 0.4; two-tailed unpaired t-test).

#### Effect of intraseptal L-733,060 in behaving animal

##### General

The observer was blinded to the pharmacological treatments in the experiments described below. L-733,060 was administered as pre-treatment, consistent with the protocol in anaesthetized animal. The antagonist was microinjected into the septum to investigate the effect of the drug on formalin-induced behaviours and septo-hippocampal theta wave activity observed concomitantly in the same animal. The behaviours monitored included nociceptive licking and flinching, and ambulation.

### Behaviour

#### Effect of microinjection of L-733,060 on nociceptive behaviours (Fig. 6a–e)

**Figure 6:**
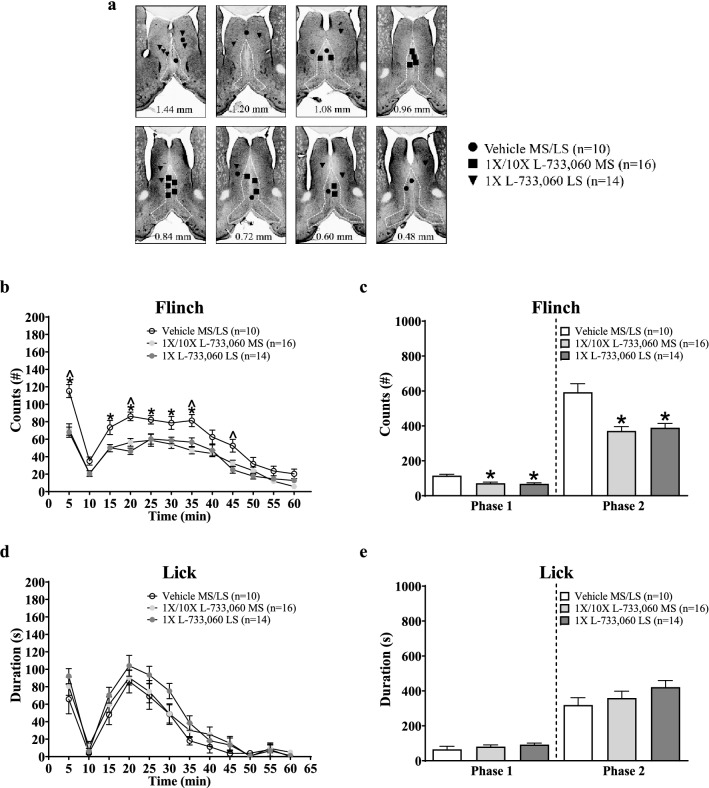
Intraseptal L-733,060 decreased nociceptive behaviours in the formalin test. In the behaving animal, formalin (1.25%, 0.1 ml) was injected into the right hind paw 15 min after microinjection of vehicle or L-733,060 into the medial (MS) or lateral (LS) septum (1 ×, 0.0176 µg/µl, MS, n = 9; LS, n = 14; 10 ×, 0.176 µg/µl, MS, n = 7). (**a**) Composite diagram of microinjection sites in the MS and LS built as in Fig. 1. (**b**, **d**) Time course graphs illustrating the biphasic (Phase 1, 1–5 min; Phase 2, 11–60 min) flinching and licking responses induced on injection of formalin. The behavioural response was binned in blocks of 5 min. (**c**, **e**) Histograms illustrating the cumulative number of flinches and cumulative duration of licking in Phases 1 and 2. Pre-treatment with L-733,060 in the MS and LS significantly attenuated formalin-induced flinching in phases 1 and 2 (**c**). However, the drug did not significantly affect licking (**e**). Data are mean ± S.E.M. Significant difference (p < 0.05): (**b**, **d**) *‘1 ×/10 × L-733,060 MS’ vs. ‘Vehicle MS/LS’ group, ^‘1 × L-733,060 LS’ groups vs. ‘Vehicle MS/LS’ group; two-way RM ANOVA followed by Bonferroni post-hoc test; (**c**) *vs. ‘Vehicle MS/LS’ group; one-way ANOVA followed by Newman–Keuls post-hoc test.

Formalin (0.1 ml, 1.25%) injection into the hind paw evoked a biphasic increase in animal agitation marked by increase in ambulation, nociceptive licking and flinching similar to that previously reported^1,37,38^. The biphasic pattern is characterized by nociceptive behavioural responses in the first 5 min after formalin injection (Phase 1) and from 11 to 60 min (Phase 2). The interphase (6–10 min) is marked by quiescence with relatively low nociceptive and ambulatory activity^1,37,38^.

The antagonist, L-733,060, was microinjected into the MS at dose of either 1 × or 10 × (Fig. 6a: 1 ×, n = 9 and 10 ×, n = 7 in MS). While, only the lower dose of the antagonist was microinjected into LS (Fig. 6a: 1 ×, n = 14). Only one dose was examined for its effect in the LS for the following reasons: (a) both 1 × and 10 × dose of L-733,060 evoked similar effects on microinjection into MS. We selected 1 × dose of the antagonist as the representative dose for evaluating the role, if any, of NK1R in LS on nociception, and (b) microinjections into LS served as ‘site’ control for microinjections into MS. There is little or no prior evidence to suggest that LS is involved in nociception or theta activation and, therefore, we did not design the study to fully develop the role of LS in nociception.

The time course of change for each of the formalin-induced nociceptive behaviours- i.e. licking and flinching- was compared between the experimental groups. Two-way RM ANOVA showed that a given formalin-induced behaviour following microinjection of 1 × or 10 × L-733,060 into the MS was similar (Treatment, F_1, 154_ = 0.98, p > 0.3 at least). Thus, data derived from the two groups were consolidated as the ‘1 ×/10 × L-733,060 MS’ group (n = 16). The remaining L-733,060 treated animals were grouped as ‘1 × L-733,060 LS’ (n = 14). The formalin-induced responses in animals microinjected with vehicle into MS (n = 6) or the LS (n = 4) were similar (Treatment, F_1, 88_ = 0.06 p > 0.8 at least, two-way RM ANOVA). Thus, two groups were also combined (‘Vehicle MS/LS’ group, n = 10).

Compared to Vehicle, microinjection of L-733,060 into both MS and LS attenuated formalin-induced flinching at various time points along the time course of the behavior (Fig. 6b; Treatment, F_2, 407_ = 17.92, p < 0.0001; two-way RM ANOVA followed by Bonferroni post-hoc test). Whereas, licking was not significantly affected (Fig. 6d; Treatment, F_2, 407_ = 2.28, p > 0.1; two-way RM ANOVA).

Consistently, phase analysis indicated that L-733,060 significantly reduced flinching in both Phase 1 and Phase 2 of the formalin test (Fig. 6c; Phase 1, Groups, F_2, 37_ = 13.21, p < 0.0001; Phase 2, Groups, F_2, 37_ = 13.46, p < 0.0001; one-way ANOVA followed by Newman–Keuls post-hoc test). Whereas, licking was not affected across both phases of the formalin test (Fig. 6e; Phase 1, Groups, F_2, 37_ = 1.16, p > 0.3; Phase 2, Groups, F_2, 37_ = 1.52, p > 0.2; one-way ANOVA).

#### Lack of effect of intraseptal L-733,060 on formalin-induced ambulation and speed

Microinjection of L-733,060 into the MS or the LS did not affect formalin-induced ambulation compared to control animals microinjected with vehicle (Treatment, F_2, 407_ = 2.32, p > 0.1; two-way RM ANOVA; data not shown). Analysis of cumulative ambulation during Phase 1 and Phase 2 also showed a lack of effect of drug treatment on ambulation on intraseptal microinjections (Phase 1, Groups, F_2, 37_ = 2.32, p > 0.1, one-way ANOVA; Phase 2, Groups, F_2, 37_ = 2.10, p > 0.1; one-way ANOVA; data not shown). The corresponding average speed (data not shown) were also not different among the groups in Phase 1 (Groups, F_2, 37_ = 0.06, p > 0.9; one-way ANOVA), Phase 2 (Groups, F_2, 36_ = 0.18, p > 0.8; one-way ANOVA) or at peak of Phase 2 (Groups, F_2, 33_ = 1 p > 0.3; one-way ANOVA).

### Theta wave activity

#### General

Theta wave activity, recorded from the hippocampal stratum radiatum and stratum lacunosum-molecular, was concomitantly monitored with behaviours during spontaneous exploration and the formalin test. The theta field activity was analysed using FFT (0.5 Hz resolution). However, the number of experiments with successful recording was less than that for behaviour, resulting in a smaller sample size for analysis (Vehicle group, n = 6, 1 ×/10 × L-733,060 MS group, n = 11, 1 × L-733,060 LS group, n = 11).

#### Effect of microinjection of L-733,060 (Fig. 7a–f)

**Figure 7:**
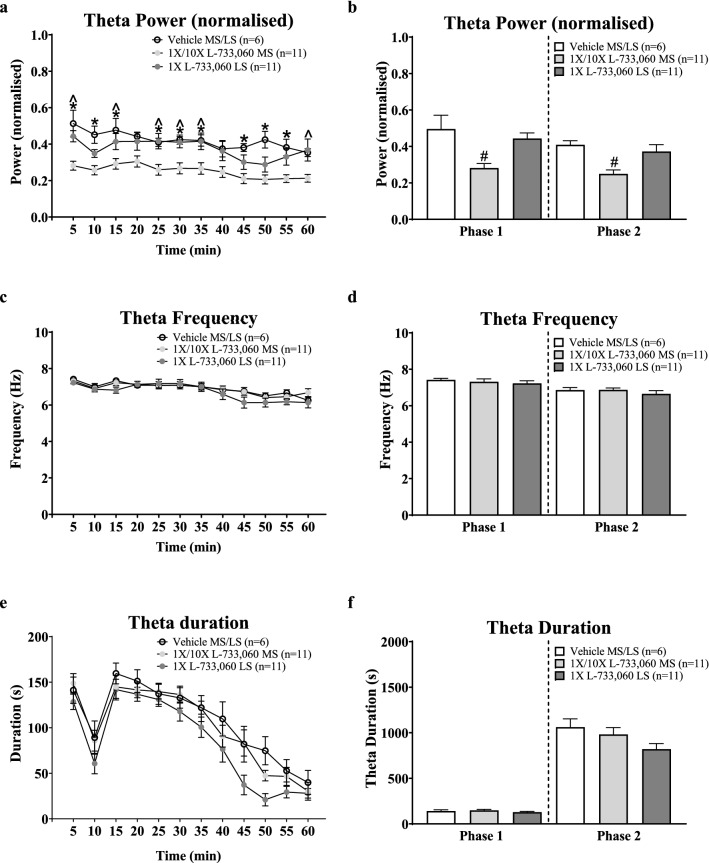
Microinjection of L-733,060 into the MS attenuated formalin-induced theta activity. Hippocampal theta activity was concomitantly recorded with formalin induced behaviour. Artifact free hippocampal theta segments of at least 2 s duration were analysed using Fast Fourier Transform (FFT; 0.5 Hz resolution) to quantify FFT theta peak frequency and power in 5 min blocks. FFT Theta peak power was normalised against the average FFT theta peak power of spontaneous theta activity recorded prior to the experiment. The duration of theta (s) reflects the cumulative duration of theta segments of at least 1 s length in blocks of 5 min. (**a**, **c**, **e**) Time course of change in theta parameters. (**b**, **d**, **f**) Histograms of the average normalised FFT theta peak power and FFT theta peak frequency, and cumulative theta duration in phases 1 and 2 of the formalin test. Administration of L-733,060 in the MS, but not LS, attenuated theta power. The drug did not evoke a significant change in FFT theta peak frequency and theta duration. Data are mean ± S.E.M. Significant difference (p < 0.05): (**a**) *‘1 ×/10 × L-733,060 MS’ group vs. ‘Vehicle MS/LS’ group and ^‘1 ×/10 × L-733,060 MS’ group vs. ‘1 × L-733,060 LS’ group; two-way RM ANOVA followed by Bonferroni post-hoc test, (**b**) ^#^vs. ‘Vehicle MS/LS’ and ‘1 × L-733,060 LS’ groups; one-way ANOVA followed by Newman–Keuls post-hoc test.

Microinjection of L-733,060 into the MS, but not the LS, evoked a significant decrease in power along the time-course of theta induced on hind paw injection of formalin (Fig. 7a; Treatment, F_2, 275_ = 8.96, p < 0.002; two-way RM ANOVA followed by Bonferroni post-hoc test). Indeed, the average normalised theta power was significantly lower in in the 1 ×/10 × L-733,060 MS group compared to vehicle pre-treated group in both Phase 1 (Fig. 7b; Groups, F_2, 25_ = 8.24, p < 0.002; one-way ANOVA followed by Newman–Keuls post-hoc test) and Phase 2 (Groups, F_2, 25_ = 7.23, p < 0.004; one-way ANOVA followed by Newman–Keuls post-hoc test).

However, pre-treatment with L-733,060 did not affect theta frequency across the time-course of theta (Fig. 7c; Treatment, F_2, 275_ = 0.68, p > 0.5; two-way RM ANOVA), and in Phase 1 (Fig. 7D; Groups, F_2, 25_ = 0.36, p > 0.7) and Phase 2 (Fig. 7d; Groups, F_2, 25_ = 0.74, p > 0.4; one-way ANOVA) of the formalin test.

In contrast to the observations in the anaesthetised animals, microinjection of L-733,060 into the MS or the LS did not significantly effect the time course of theta duration induced on injection of formalin in the awake animal (Fig. 7e; Treatment, F_2, 275_ = 1.12, p > 0.3; two-way RM ANOVA). Consistently, the durations of theta in Phase 1 and Phase 2 were similar across groups (Fig. 7f; Phase 1, Groups, F_2, 25_ = 1.09, p > 0.3; Phase 2, Groups, F_2, 25_ = 2.60, p > 0.09; one-way ANOVA).

#### Lack of effect of L-733,060 on exploratory theta

Following drug microinjection, the animal was allowed to explore the familiar test chamber for 15 min prior to hind paw formalin injection. The average power of theta wave activity observed during exploration by the animal in the period before and after microinjection was compared to determine the effect of L-733,060. The power of exploratory theta before vs. after microinjection was similar in both vehicle (0.10 ± 0.02 mV^2^ vs. 0.10 ± 0.02 mV^2^ (n = 5), p > 0.7; two-tailed unpaired t-test) and L-733,060 microinjected animals (MS: 0.09 ± 0.02 mV^2^ vs. 0.08 ± 0.02 mV^2^ (n = 8), p > 0.7; LS: 0.11 ± 0.02 mV^2^ vs. 0.10 ± 0.02 mV^2^ (n = 10), p > 0.7; two-tailed unpaired t-test).

The number of animals in each group analysed above differs from that after formalin injection into the right hind paw as the EEG recordings of a few animals following microinjection were not recorded due to technical issues. Interestingly, the normalised formalin-induced theta power was about half of the exploratory theta (see Fig. 7a).

## Discussion

The present study has led to key novel findings. One, peptide neurotransmission at NK1R, presumably involving SP in MS, modulates nociceptive responses in septo-hippocampus. Thus, on one hand, microinjection of NK1R agonist, SP, into MS evoked a robust suppression of the CA1 PS that was attenuated by intraseptal microinjection of NK1R antagonist, L-733,060. The SP-induced suppression was antagonized by systemic atropine which is consistent with the idea that SP evoked the suppression via NK1R-mediated excitation of septal cholinergic neuron. On the other hand, intraseptal L-733,060 attenuated formalin-induced suppression of CA1 PS and duration of theta activity in anaesthetized animal while attenuating formalin-induced theta power in behaving animal. Notably, theta activation and suppression of CA1 PS are parts of spectrum of neural responses involved in encoding of information to salient stimulus, including to formalin injection that are attenuated on destruction of septal cholinergic neurons^3,5,39^.

Interestingly, L-733,060 selectively affected response to noxious stimulus. Thus, while intraseptal L-733,060 antagonized formalin-evoked responses, the drug failed to antagonize intraseptal carbachol-induced theta activation and the suppression of CA1 PS, and the theta induced on exploration of familiar environment suggesting that NK1R mechanisms are not pivotal across all forms of septo-hippocampal network activation.

The theta activity in anaesthetized rat was recorded from the pyramidal cell layer that reflects an integration of theta-rhythmic voltage changes across the cell body and proximal dendrites of pyramidal cells that are driven partly by septal cholinergic neurons^21,40–42^. Since the NKR are located almost exclusively on cholinergic neurons, the findings suggest that microinjection of L-733,060 antagonized peptidergic mediated activation of medial septal cholinergic neurons leading to a loss of theta wave activity in the hippocampal pyramidal cell region. This is also consistent with observation that the antagonist strongly attenuated formalin-induced suppression of CA1 PS, which is also mediated by cholinergic neurons in the MS^5^.

Further, in behaving animal, microinjection of L-733,060 into MS attenuated power of formalin-induced theta wave activity recorded from dendritic regions of CA1 pyramidal cells. The power of dendritic theta in behaving animal reflects an integration of voltage changes across somatic-dendritic dipoles^21,40–42^. Intraseptal L-733,060 likely affected power in awake animal through modulation of the somatic component (see above). Conversely, a decrease in the power observed in behaving animal suggests that the loss of theta in anaesthetized animal is due to loss of power of the L-733,060 sensitive theta, the residual theta being relatively insensitive to L-733,060. Collectively, the foregoing suggest NK1R mediates, at least in part, the septo-hippocampal nociceptive response to hind paw injection of formalin across both anaesthetized and awake animals.

Two, NK1R-mediated peptidergic mechanisms in MS modulate aspect of nociceptive behaviours. In this context, microinjection of L-733,060 into the MS attenuated flinching in awake rat. Interestingly, the flinching was partially attenuated while licking was not significantly affected. Likewise, intraseptal L-733,060 evoked only a partial decrease in the power of formalin-induced septo-hippocampal theta. Increasing the dose of intraseptal L-733,060 by tenfold did not amplify the pattern of behavioural and electrophysiological changes vis-à-vis the lower dose of L-733,060. However, the study did not evaluate another higher dose of L-733,060 for an effect on nociception. Considering this, a parsimonious view is that intraseptal mechanisms that mediate nociceptive flinching, rather than licking are more sensitive to modulation by NK1R. Alternately or conjointly, the partial effect of L-733,060 on nociception might reflect a limited role of the NK1R in nociception due to the circumscribed localization of the NK1Rs on only the cholinergic neurons in the MS. Here, it is notable that the septal mechanism mediating both septo-hippocampal theta activation and nociception are multifactorial and involve intraseptal cholinergic, glutamatergic, and GABAergic mechanisms^1,16,29,33,41–44,48,58–61^. Indeed, in comparison to L-733,060, pharmacological manipulations that attenuate the excitatory glutamatergic transmission in MS attenuate both nociceptive flinching and licking in the formalin test^24^. Glutamate and glutamate receptors are relatively ubiquitous in MS as compared to NK1Rs.

The behavioural and electrophysiological effects with intraseptal L-733,060, especially at peaks of the formalin test, were observed with no significant change in ambulation. Speed of ambulation was also unaltered on microinjections. The dissociation indicates that anti-nociception and electrophysiological changes evoked on intraseptal microinjection of L-733,060 were not secondary to ambulatory changes or sedation.

Interestingly, microinjection of L-733,060 into LS also selectively attenuated nociceptive flinching. Even though microinjection of the drug into either MS or LS equally decreased flinching behaviour, only microinjection into MS decreased theta activation. The selective effect on theta with microinjection into MS is consistent with the role of the region as a gateway for hippocampal theta generation. The electrophysiological effects, being observed with microinjection into MS but not LS, point to localization of drug effect to the region of microinjection. This, then, suggests that the peptide neurotransmission at NK1R is a modulator of acute affective-motivational behaviours in both the MS and the LS. Consistent with a shared role of NKRs in affect-motivation along the medio-lateral axis of the septum, it is notable that SP is released, or its tissue levels increase in LS and MS under stressful conditions^43,44^. A common peptidergic input and/or intraseptal peptidergic connections may play a role in the coordinate response of MS and LS. Especially, the LS presents a rich tapestry of SP afferent fibres^45–47^, while the dorsal and intermediate regions of LS that contain SP positive neurons also send afferent to MS region^45–47^.

**Figure 8:**
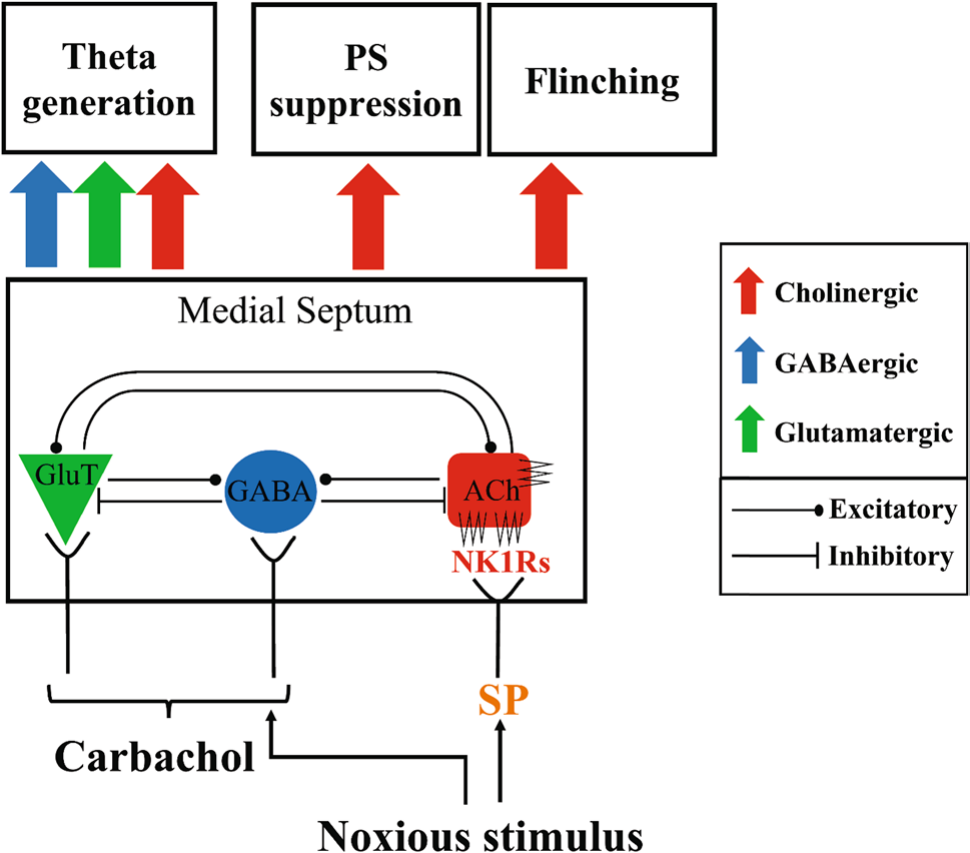
A model of NK1R-mediated nociception in the formalin test. The diagram depicts septal cholinergic neurons (Ach) which are interlinked with the local non-cholinergic neurons, namely GABAergic (GABA) and glutamatergic (GluT) neurons^66,67^. Pertinent here is that the neurokinin NK1Rs are localized to the cholinergic neurons in the region. The present study suggests the septal NK1Rs modulate formalin nociception. For instance, SP, an agonist at NK1Rs, and injection of formalin equally evoke a suppression of CA1 PS that is antagonized by intraseptal L-733,060, an antagonist at NK1Rs. Likely, the suppression of CA1 PS is linked to direct excitation of the septo-hippocampal cholinergic neurons. In this context, formalin injection evokes a release of acetylcholine in hippocampus which is implicated in suppression of CA1 PS^5,20^. Interestingly, while the power of formalin-induced theta is attenuated by intraseptal L-33,060, intraseptal SP evokes little or no theta activation even though the agonist suppressed the CA1 PS. This suggests that NK1R mediation of formalin theta is not predicated entirely on release of acetylcholine in hippocampus. The cholinergic mediation of theta is suggested to be mediated partly through intraseptal release of acetylcholine that modulates septal non-cholinergic neurons and affect power of theta^5,41–44,48^. Potentially, the NK1R modulation of power of formalin theta might be mediated through similar circuit mechanism involving the septal cholinergic and non-cholinergic neurons. However, the NK1R modulation of theta activation is nociceptive specific since antagonizing septal NK1R does not affect exploratory theta or intraseptal carbachol induced theta activation. Interestingly, the non-cholinergic neurons are also implicated in generation of theta rhythm to intraseptal carbachol^5,16,43,45–47,61^. Finally, while formalin-induced electrophysiological changes and flinching are modulated by septal NK1Rs, it is unclear if the two also share a common pool of cholinergic neurons and/or similar circuit mechanisms.

Strikingly, our findings indicate that intraseptal SP evoke little theta activation, even though the drug evoked a robust suppression of the PS. In contrast, microinjection of carbachol into MS elicited a relatively robust theta activation. Notably, carbachol also evoked a suppression of the CA1 PS that was quite like that seen with intraseptal SP. The foregoing comparison suggests that the activation of NK1Rs does not drive theta activation per se, which is consistent with the suggestion above that the NK1Rs are not pivotal for theta activation. Nonetheless, NK1Rs modulates nociceptive theta activation since intraseptal L-733,060 attenuated the power of formalin-induced theta, though the power of theta evoked on exploration was not affected. This suggests a selective role of NK1Rs during nociception. Figure 8 brings out the potential intraseptal circuit mechanisms that underpin the nociceptive effects of NK1R, suggesting that the multiplicity of effects involving septal NK1R involves different circuit mechanisms, at least in part.

In summary, the novel observations reported here show that NKRs, especially NK1Rs in MS are mediators of an aversive valence affecting formalin-induced overt nociceptive behaviour and the nociceptive processing in the septo-hippocampus. The current findings, showing that septal NK1Rs mediate nociception, seen in juxtaposition with pro-nociceptive role of NKRs in the spinal cord and the brainstem^36,48,49^, suggest a common role of the NKRs in mediating nociceptive processing along the neural axis in CNS. In MS, the neurotransmission at NK1Rs may modulate septo-cingulate and septo-hippocampal cholinergic neurons and co-ordinately influence different aspects of nociception. For example, the septo-cingulate neurons are known to mediate nociceptive behaviours, while the septo-hippocampal cholinergic neurons are implicated in formalin-induced theta activation and nociception^3,5,20,50^. Further, broadly, the forebrain cholinergic neurons and the cholinergic transmission are implicated in nociception, affecting both nociception and anti-nociception^51–53^.

## Materials and methods

### Animals

The experimental studies were performed in accordance with the relevant guidelines and regulations approved by the local institutional Animal Care and Use Committee (IACUC) from National University of Singapore (NUS), and in compliance with ARRIVE guidelines. Experiments were performed on adult male Sprague Dawley rats aged 7–8 weeks old (270 to 350 g) purchased from InVivos Pte Ltd. Animals were housed under controlled temperature and humidity in the allocated animal facility with water and pelleted food ad libitum under a 12 h dark/light cycle. Allocation to experimental groups were randomised.

### Surgical procedures

#### Anaesthetized animals

Procedures are in accordance with those described previously^3^. Urethane (1 g/kg, i.p.; Sigma, USA) anaesthetized animals were mounted onto a stereotaxic frame (Stoelting Co, USA). Drugs were microinjected either via a 36G microinjection needle coupled to an Exmire microsyringe (Ito Corporation, Japan) or a double-barreled cannula. These were lowered through burr holes made in the region overlying the medial septum (MS) (A0.5 mm from Bregma, L0.5 mm from midline, and V6.5 mm from the cortical surface^54^). The double-barreled cannula was fashioned by fusing two silicon tubes (34G, 100 um external diameter, WPI, USA) and attaching one end to a microsyringe via polythene tubing.

A bone flap was made over the left cerebral hemisphere for lowering of stimulating and recording electrodes^37^ into the hippocampus. A concentric bipolar stimulating electrode (Model NE-100, David Kopf, USA) was placed in the left hippocampal field CA3 (P3.0 mm from Bregma, L2.4 mm from midline, and V4.0 mm from the cortical surface^54^), while a saline-filled carbon fiber glass recording electrode was directed towards the pyramidal cell layer of the left hippocampal field CA1 (P3.6 mm from Bregma, L2.0 mm from midline, and V4.0 mm from the cortical surface^54^), oriented at an angle of 5° to the vertical from the right.

#### Cortical implants

Survival surgery was performed under aseptic conditions using stereotaxic technique as previously described^1,2,37^. Briefly, anesthesia was induced and maintained with 5% and 2% isoflurane, respectively, with oxygen flow at 1 L/min. A single barreled 26G stainless steel guide cannula (Plastic One, Roanoke, VA, USA) was directed towards the MS (A0.5 mm from Bregma, 0.0 mm from midline and V5.8 mm from the cortical surface ^54^). Animals were also implanted with a depth recording electrode, constructed by twisting a pair of stainless-steel Teflon-insulated wires (125 µm in diameter; AM Systems, USA) and connected to gold plated male pins at one end. The recording electrode was directed towards the stratum radiatum of the left hippocampal field CA1 (P3.0 mm to Bregma, L2.4 mm from midline, and V3.0 mm from the cortical surface^54^). Implants were secured with support screws and dental cement. Following surgical procedure, the animals were housed individually until further experimentation.

Post-operatively, the animals recovered for at least 7 days, during which they were administered the analgesic buprenorphine (0.03 mg/kg for 3 days, i.p.) and the antibiotic enrofloxacin (10 mg/kg for 5 days, i.p.).

### In vivo intracerebral stimulation and electrophysiological recording

#### Anaesthetized animals

Hippocampal field CA1 was identified by the observation of complex spike activity or population spike (PS). PS was generated by stimulation of the ipsilateral CA3 region (0.1 Hz, 0.01 s pulse duration) through a constant current stimulation isolation unit (Grass S88 stimulator; Grass Technologies, USA). CA3 stimulation intensity was adjusted to generate a PS amplitude at 70% of the maximum under large irregular field activity^37,55^. Signal recorded by the glass electrode were split to record hippocampal field activity (band passed at 1–100 Hz, digitized at 256 Hz) and PS (band passed 1–3000 Hz, digitized at 10 kHz).

#### Behaving animals

Hippocampal field activity (band passed at 1–100 Hz, digitized at 256 Hz) in the behaving animals was recorded via a flexible recording wire connected to the implanted head stage on the animal. At the other end, the wire was connected to an amplifier via a commutator.

### Drugs, microinjection procedure and hind paw injection of formalin

#### Drugs

The drugs used were (i) the NK1R agonist, Substance P (SP; 1 and 2 μg/μl; #S6883, Sigma, USA), (ii) the NK1R antagonist, L-733,060 (1 ×: 0.0176 µg/µl and  × 0.176 µg/µl; #1145, Tocris Bioscience, UK) and (iii) the cholinergic receptor agonist, carbamoylcholine chloride (carbachol; 0.156 μg/μl; #C4382, Sigma, USA).

The functional effects of different doses of SP and L-733,060 were explored in this study. Notably, 1 × concentration of L-733,060 in the present study is anti-nociceptive on microinjection into the rostral ventral medulla^48^. While we initially used the lower dose of L-733,060, arising from the results, especially in behaving animals, we also examined the effect of the higher dose of L-733,060 to explore whether the different aspects of nociception are differentially sensitive to antagonism by the antagonist. The concentration of carbachol used in the experiments is based on published work^65^.

#### Microinjection

Drugs were microinjected, rather than administered systemically, so as to understand the role of local, i.e. septal, NK1Rs in nociception. A systemically administered drug is expected to evoke an effect that is an aggregate of the manifold functional outcomes, some of which may be in opposition due to the actions of the drug at its receptors at different level of neural axis. Such an effect may mask localized drug actions. Thus, for example, SP evokes antinociception given systemically, which is in contrast to the nociceptive response evoked following intrathecal administration of the drug^56–59^.

In the present study, the microinjection volume for all experiments were 0.5 µl. All drugs were dissolved in saline (0.9% w/v sodium chloride; Sigma, USA) containing the Alcian blue dye (0.5% w/v; Sigma, USA). The dye solution was administered as the corresponding vehicle. All microinjections were carried out in a blinded fashion. The following procedures were followed during microinjection in anaesthetized and behaving animals.

##### Microinjection in anaesthetized animals

The microinjection needle/cannula was lowered into the MS at an angle of 5° to the right from the vertical. The double barrel was orientated along the anterior–posterior axis of the MS. The antagonist, L-733,060, or vehicle was microinjected via the anterior cannula while SP or carbachol was microinjected via the posterior cannula.

##### Microinjection in behaving animals

Drugs were administered via a 33G stainless steel internal cannula (Plastic One, Roanoke, VA, USA) connected to a microsyringe (Hamilton, USA) by a polyethylene cannula connector assembly system. The internal cannula protrudes from the tip of the guide cannula by 1.0 mm. With gentle restraint, the internal cannula was inserted into the implanted guide cannula and the drug was microinjected over a period of 30 s. The internal cannula was left in situ for at least 1 min to allow diffusion and to minimize backflow. Drugs were administered in a blinded fashion.

#### Hind paw injection of formalin

Formalin was injected subcutaneously (intra-plantar) into the right hind paw in both anaesthetized (5% formalin) and behaving (1.25% formalin) animals. The concentrations selected are within the formalin concentration range that evokes concentration-dependent biphasic increase in animal nociceptive behaviors, with the lower concentration being submaximal while the higher concentration being at the upper end of the concentration range^18,60–62^. The lower concentration was used in behaving animals so as to reduce distress experienced by the animals. Nonetheless, this concentration evokes robust effect on CA1 neural processing in behaving animals^38,61^. The higher concentration (5%), similarly, evokes robust neural effects in anaesthetized animal^3^.

Further, formalin, and not brief thermal or mechanical stimuli was used as a noxious stimulus in the present study because MS modulates formalin-induced persistent behaviors, but not reflexive nociceptive behaviors to peripheral mechanical and thermal stimuli applied to naïve animals^2,37^. Indeed, the formalin test is widely used to investigate physiological and pharmacological mechanisms of overt nociceptive behaviors, these behaviors being relatively well characterized^16^. Moreover, peripheral hypersensitivity is not widely explored in this model, in part because formalin injection induces hypoalgesia to thermal and mechanical stimuli at site of injection while evoking hyperalgesia elsewhere on body surface^63^. The basis of such effects, which are somewhat paradoxical, are not well studied or understood.

### Histology

At the end of each experiment, the animals were given an overdose of urethane (1.5 g/kg, i.p, Sigma, USA) and perfused transcardially with 0.9% sodium chloride solution followed by 10% formalin (Merck, Germany). The brain was removed and placed in the fixative. 100 µm coronal sections were made on a vibratome (Leica VT1200, Leica Microsystems GmbH, Wetzlar, Germany) and collected in Tris-buffered saline (TBS). Sections through the stimulating, recording and microinjection sites were collected for Nissl stain with 0.5% w/v Cresyl violet (Sigma, USA).

### Experimental protocol

#### Dose-dependent effect of SP

This experiment was performed to investigate the effect of intraseptal microinjection of NK1R agonist, Substance P (SP; 1 or 2 μg/μl, 0.5 µl), on the amplitude of CA1 PS and theta wave activity. The agonist, or the corresponding vehicle, was microinjected after recording control PS for a period of 2 min during period of large irregular field activity (LIA). Following microinjection, hippocampal neural responses were recorded continuously for 20 min and again for 2 min at the 40th and 60th min after microinjection. The microinjection was repeated three times (SP1-3), the microinjections being at least 1 h apart.

#### Effect of the cholinergic muscarinic receptor antagonist, atropine, on SP-induced responses

Previously, the laboratory has demonstrated that septal cholinergic neurons modulate the amplitude of CA1 PS and theta wave activity^5^. Further, NK1Rs in MS are expressed almost exclusively on cholinergic neurons in the region^25^. Thus, this experiment was performed to investigate whether the electrophysiological effects of intraseptal SP are antagonized by atropine (5 mg/kg, i.p.). The protocol is as described immediately above with the following variations: (a) SP microinjection was repeated four times (SP1–4), the microinjections being at least 1 h apart, (b) vehicle or atropine was administered intraperitoneally 30 min before intraseptal SP. The intraperitoneal administration involved the following combinations of injections—vehicle injections prior to SP2 and SP3, or vehicle prior to SP2 and atropine prior to SP3.

#### Selectivity of antagonism by the NK1R antagonist L-733,060

Different concentrations of L-733,060 (1 × or 10 ×) were microinjected into the MS in separate experiments to investigate the ability of the drug to antagonize hippocampal responses evoked on intraseptal microinjection of either SP (2 μg/μl) or carbachol (0.156 μg/μl). Each experiment comprised of microinjections of the SP (SP1-3) or carbachol (C1–3) at least 60 min apart. In addition, L-733,060 or vehicle was microinjected only once, 15 min before SP2 or C2, under condition of LIA. Because the second microinjection of the agonist was preceded by microinjection of the antagonist or vehicle, the data set was labelled as either L-733,060 + SP2, L-733,060 + C2, Vehicle + SP2 or Vehicle + C2. The effects of agonists were monitored continually for 20 min after microinjection and again for 2 min at the 40th and 60th min.

#### Effect of intraseptal L-733,060 on formalin-induced nociception

The effect of L-733,060 on formalin induced hippocampal responses was first characterised in the anaesthetised animal. L-733,060 or vehicle was microinjected into septum 15 min preceding intra-plantar injection of formalin (5%, 0.05 ml) into right hind paw. The time of formalin injection was taken as 0 min. Hippocampal responses to formalin were continually recorded for 20 min after formalin injection and again for 2 min at the 40th and 60th min after injection.

#### Effect of intraseptal L-733,060 on formalin-induced nociceptive responses in behaving animal

The formalin test was conducted in a test chamber (L43.2 cm × W21.7 cm × H30.5 cm; model ENV-515, Med Associates Inc., USA). Animals were habituated to the experimental chamber for 3 consecutive days for at least 60 min each day.

Prior to the experiment, control hippocampal theta wave activity was recorded for at least 10 min during exploration of the test arena by the animal. The power of exploratory theta was used to normalize the power of theta induced following injection of formalin. The exploration of the test arena was followed by microinjection of either L-733,060 (1 × or 10 ×) or vehicle. The formalin test was performed 15 min after microinjection of L733,060 or the associated vehicle. The 15 min period before formalin injection is labelled as the baseline recording during which the animal was allowed to spontaneously explore the test chamber. The formalin test involved injecting formalin (1.25%, 0.1 ml) subcutaneously into the plantar surface of the right hind paw.

Formalin-induced nociceptive responses i.e., hippocampal field activity, flinching and licking of the injected paw, were recorded for 60 min. The number of paw flinching and duration of licking of the injured paw were quantified in 1 min blocks. In addition, formalin-induced ambulation (ambulatory distance) was also measured by the activity monitor Actimot (Med Associates Inc., USA). An ambulatory episode was signalled when the distance travelled by the animal exceeded the pre-set space of 4 × 4 IR beams (approximately 10 cm × 10 cm). The speed was measured by the software if the animal moved for more than 2 s.

### Data analyses

#### Electrophysiological recording

PS and hippocampal theta activity were analysed offline using the Spike 2 software. PS amplitude (mV) was calculated as the average amplitude between the negative peak from the 2 positive peaks around it as described previously^3,37,55,64,65^. PS amplitude was averaged over six sweeps in 1 min blocks. The magnitude of PS amplitude reflects the size of the neuronal population discharging synchronously in response to CA3 stimulation.

Hippocampal field activity was digitally filtered at 1–40 Hz with finite impulse response (FIR) filter. Artifact free theta segments of at least 2 s duration were subjected to fast Fourier transform (FFT) analyses (frequency resolution of 0.5 Hz) in 1- or 5-min blocks to derive the average FFT theta peak frequency (Hz; 3–6 Hz in anaesthetized animals, 4–12 Hz in behaving animals) and FFT theta peak power (mV^2^) which is the peak power in the theta frequency range. The computed FFT theta peak power was normalized against the average FFT theta peak power of spontaneous theta activity recorded prior to the drug microinjection (in anaesthetized animals) or during exploration of test arena prior to the formalin test (in behaving animals). Duration of theta was calculated by computing the period of time (s per 1 or 5 min block) for which theta was visually identified as a continuous sinusoidal oscillation of at least 1 s duration at frequencies of 3–12 Hz.

#### Formalin-induced responses

The total ambulatory distance (cm) and average speed (cm/s) in 5-min blocks was extracted from the Actimot software. However, in some instances, ambulatory movements did not reach the threshold for calculating speed. Such points were not included in analysis of speed. Formalin-induced flinches (counts) and duration of licking (s) of the injured paw were summed in 5 min blocks. The cumulative ambulatory distance, flinching or licking were computed for phase analysis in phase 1 (1–5 min) or Phase 2 (11–60 min).

### Statistical analysis

Statistical analysis of data was carried out using Prism 4 (GraphPad Software, USA). The time course graphs depicting the changes in the electrophysiological or behavioural parameters to a single treatment were analysed using one-way repeated measure (RM) ANOVA followed by Newman–Keuls post-hoc test. The changes in the time course across multiple treatments were compared with two-way RM ANOVA followed by Bonferroni post-hoc test. The differences between means of multiple groups were analysed using one-way ANOVA followed by Newman–Keuls post-hoc test. In instances when the Bartlett’s test showed unequal variance, the data was normalized by logarithmic transformation. Otherwise, the non-parametric Kruskal–Wallis test was applied to the data. Differences were statistically significant at p ≤ 0.05. The data are mean ± standard error of mean (S.E.M).
